# Proteome‐minimized outer membrane vesicles from *Escherichia coli* as a generalized vaccine platform

**DOI:** 10.1002/jev2.12066

**Published:** 2021-02-16

**Authors:** Ilaria Zanella, Enrico König, Michele Tomasi, Assunta Gagliardi, Luca Frattini, Laura Fantappiè, Carmela Irene, Francesca Zerbini, Elena Caproni, Samine J. Isaac, Martina Grigolato, Riccardo Corbellari, Silvia Valensin, Ilaria Ferlenghi, Fabiola Giusti, Luca Bini, Yaqoub Ashhab, Alberto Grandi, Guido Grandi

**Affiliations:** ^1^ Department of Cellular, Computational and Integrative Biology (CIBIO) Laboratory of Synthetic and Structural Vaccinology University of Trento Trento Italy; ^2^ Toscana Life Sciences Foundation Siena Italy; ^3^ GlaxoSmithKline Vaccines Siena Italy; ^4^ Department of Life Sciences Functional Proteomics Laboratories University of Siena Siena Italy; ^5^ Palestine‐Korea Biotechnology Center Palestine Polytechnic University Hebron Palestine; ^6^ BiOMViS Srl Siena Italy

**Keywords:** cancer, infectious diseases, outer membrane vesicles (OMVs), synthetic biology, vaccines

## Abstract

Because of their potent adjuvanticity, ease of manipulation and simplicity of production Gram‐negative Outer Membrane Vesicles OMVs have the potential to become a highly effective vaccine platform. However, some optimization is required, including the reduction of the number of endogenous proteins, the increase of the loading capacity with respect to heterologous antigens, the enhancement of productivity in terms of number of vesicles per culture volume. In this work we describe the use of Synthetic Biology to create *Escherichia coli* BL21(DE3)Δ60, a strain releasing OMVs (OMVs_Δ60_) deprived of 59 endogenous proteins. The strain produces large quantities of vesicles (> 40 mg/L under laboratory conditions), which can accommodate recombinant proteins to a level ranging from 5% to 30% of total OMV proteins. Moreover, also thanks to the absence of immune responses toward the inactivated endogenous proteins, OMVs_Δ60_ decorated with heterologous antigens/epitopes elicit elevated antigens/epitopes‐specific antibody titers and high frequencies of epitope‐specific IFN‐γ‐producing CD8^+^ T cells. Altogether, we believe that *E. coli* BL21(DE3)Δ60 have the potential to become a workhorse factory for novel OMV‐based vaccines.

## 1 INTRODUCTION

More than 60 years ago, it was accidentally discovered that Gram‐negative bacteria release Outer Membrane Vesicles (OMVs) (Bishop & Work, 1965; Chatterjee & Das, 1967). As often happened in science, the discovery remained almost unnoticed for several years and only recently, scientists became aware of the amazing aspects of the OMV biology and of their potential for biotechnological applications. OMVs are closed spheroid particles of heterogeneous size, 50–300 nm in diameter, generated through a ‘budding out’ of the bacterial outer membrane. They represent a distinct secretory pathway with a multitude of functions, including inter and intra species cell‐to‐cell cross‐talk, biofilm formation, genetic transformation, defence against host immune responses, and toxin and virulence factor delivery to host cells (Ellis & Kuehn, 2010; Kulp & Kuehn, 2010).

OMVs are becoming an attractive vaccine platform for their excellent adjuvanticity (Chen et al., 2010; Ellis & Kuehn, 2010), the possibility of decorating them with heterologous antigens (Gerritzen et al., 2017; Irene et al., 2019; Kesty & Kuehn, 2004), and the simplicity of their production and purification process (Berlanda Scorza et al., 2012). Indeed, OMV‐based vaccines have already reached the market (Ladhani et al., 2016; Serruto et al., 2012), while others are in advanced clinical phases (Gerke et al., 2015; Rossi et al., 2016).

OMV‐based vaccines can be classified in two categories. The first one includes vaccines constituted by OMVs directly purified from the pathogen of interest (Kaparakis‐Liaskos & Ferrero, 2015; Van Der Pol et al., 2015). The rationale of developing such vaccines is that OMVs provide both the immune stimulatory molecules (adjuvants) and the protective antigens. The prototype of this category is Bexsero, the Meningococcal Group B vaccine, which, in addition to purified meningococcal antigens, is formulated with vesicles from the *Neisseria meningitidis* New Zealand strain (Parikh et al., 2016). The second category includes OMVs decorated with heterologous antigens and obtained from a properly engineered Gram‐negative bacterial species (Kaparakis‐Liaskos & Ferrero, 2015; Van Der Pol et al., 2015). Such vaccines exploit the excellent adjuvanticity properties of OMVs and have the advantage to potentially target any kind of bacteria, viruses and cancer, provided that specific protective antigens are selected.

No vaccines belonging to the second category are currently available for humans. This is in part because for its full‐blown development, the OMV platform still needs to be optimized. Such optimization should include the development of efficient strategies for OMV engineering, the modulation of the OMV adjuvanticity to avoid potential reactogenicity, and the elimination of unnecessary OMV endogenous proteins.

Here, we describe the application of Synthetic Biology for the construction of a novel *E. coli* strain, named *E. coli* BL21(DE3)*Δ60*, releasing OMVs (OMVs*_Δ60_*) deprived of 59 endogenous proteins. The rationale for creating such strain is threefold. First, we hypothesized that by eliminating OMV endogenous proteins we could increase the loading capacity of OMVs with respect to recombinant antigens. Second, we predicted that heterologous antigens expressed in proteome‐minimized OMVs should be more immunogenic because of their increased amount in OMVs and of the absence of endogenous proteins that could dilute the antigen‐specific immune responses. Third, in view of human applications, the availability of OMVs carrying a reduced number of irrelevant proteins should offer an important additional advantage. As predicted, we show that OMVs*_Δ60_* have a number of important features, which ultimately lead to the elicitation of potent antibodies and T cell responses against heterologous antigens. Overall, our data indicate that E. *coli* BL21(DE3)*Δ60* is a particularly useful strain for the development of effective vaccines against infectious diseases and cancer.

## 2 MATERIALS AND METHODS

### 2.1 Bioinformatics analysis and software

The *E. coli* BL21(DE3) proteome was analyzed to predict membrane‐associated and periplasmic proteins. A meta‐prediction pipeline that employs different subcellular localization prediction tools in three phases of positive and negative selections was used (Hisham & Ashhab, 2018). The identified proteins were subsequently assessed for their biological function and classified (essential *vs*. non‐essential) according to the ‘Keio collection’ (Baba et al., 2006), and EcoCyc *E. coli* database (Keseler et al., 2017), respectively. Oligonucleotides for guide DNAs, donor DNAs and primers were designed using Serial Cloner and Benchlink and BLAST was used for alignments (Altschul et al., 1990). Densitometry was performed with Image Studio Lite Version 5.2 (Li‐Cor Biosciences, Lincoln, NE USA) and for 2‐DE map analysis, ImageMaster 2D Platinum v.6.0 software was used (GE Healthcare, Chicago, IL USA).

### 2.2 Bacterial strains, culture conditions and plasmids

*E. coli* strains (BL21(DE3) derivatives, DH5α, TOP10) were routinely cultured in lysogeny broth (LB Miller; Merck, Darmstadt, Germany) supplemented with 100 mg/L ampicillin (Carl Roth, Karlsruhe, Germany), 25 mg/L chloramphenicol (Merck, Darmstadt, Germany), 50 mg/L kanamycin (Thermo Fisher Scientific, Waltham, MA USA) or a combination of them, where necessary. For recombinant protein expression in OMVs, cultures were supplemented with 0.1 mM isopropyl‐β‐D‐thiogalactopyranoside (IPTG, Merck, Darmstadt, Germany) at OD_600_ between 0.5 and 0.7 and OMVs were collected after 4 h from induction. Fermentation was carried out in an EZ control bioreactor (Applikon Biotechnology, Schiedam, Netherlands) at 30°C until OD_600_ 0.5, then growth continued at 25°C, pH 6.8 (±0.2), dO2 > 30%, 280–500 rpm. At OD_600_ 1.0, the culture was induced with 0.1 mM IPTG together with a feed consisting in ampicillin (50 mg/L) or chloramphenicol (12.5 mg/L), glycerol (15 g/L), and MgSO_4_ (0.25 g/L).

Plasmids encoding FhuD2, SpA_KKAA_, Csa1, Hla_H35L_, FhuD2‐mFAT1 and Nm‐NHBA were already described (Fantappiè et al., 2017; Grandi et al., 2018; Irene et al., 2019). For the construction of plasmids encoding ClfA_Y338A_, FhuD2‐Bp, FhuD2‐OVA and FhuD2‐hFAT1, the PIPE‐PCR method was used (Klock & Lesley, 2009). Briefly, the coding sequences were chemically synthetized (Geneart, Thermo Fisher Scientific, Waltham, MA USA) and PCR amplified or several oligonucleotides were assembled stepwise (in case of Bp) using the primers reported in Table S3. In parallel, a pET21b+ derivatives carrying either the sequence encoding the leader peptide for secretion of *E. coli* Lpp (pET‐Lpp plasmid) or Lpp‐FhuD2 (for epitope fusions) (Irene et al., 2019) were linearized using the primer couples Lpp‐R‐plasmid/nohisflag and Lpp‐FhuD2‐R‐plasmid/nohisflag, respectively (Table S3). The PCR products were digested with DpnI (New England Biolabs, Ipswich, MA USA), combined (2 μL each) and used to transform *E. coli* HK100 chemically competent cells. Positive clones identified by colony PCR using GoTaq Green Master Mix (Promega, Madison, WI USA) and gene specific primer (Table S3) were subjected to sequence analysis (Eurofins Scientific, Luxemburg) before using the resultant plasmids (Table S3) for transformation of OMV producer strains.

### 2.3 Construction of *E. coli* BL21(DE3)*Δ60*

Gene deletions were accomplished using our previously established CRISPR/Cas9‐based genome editing protocol (Zerbini et al., 2017). The preparation of the 90 pCRISPR‐SacB‐gDNA plasmids were done using double‐stranded synthetic oligonucleotides (Merck, Darmstadt, Germany) encoding for the guide RNA reported in Table S4. Stepwise single gene mutagenesis was achieved by co‐transformation of electrocompetent cells of the recipient strain (*E. coli* BL21(DE3)*ΔompA*_pCasRed, *E. coli Δ01*_pCasRed, etc.) with the gene‐specific pCRISPR‐SacB‐gDNA and its respective double‐stranded donor DNA (ds‐dDNA) (Table S5). Gene‐specific primer pairs used for screening of colonies with GoTaq Green Master Mix (Promega, Madison, WI USA) are listed in Table S6. Genomic DNA was extracted from *E. coli* BL21(DE3)*ΔompA* and *E. coli* BL21(DE3)*Δ58* using PowerSoil DNA Isolation Kit (Mo Bio Laboratories, Carlsbad, CA USA) and the genomes were fully sequenced. Briefly, libraries were prepared using the Nextera XT DNA Library Preparation Kit (Illumina, San Diego, CA USA), and library quality was assessed using the Caliper LabChip GX (High‐Throughput Bioanalyzer) following manufacturers guidelines. Sequencing was performed on the HiSeq 2500 system (Illumina, San Diego, CA USA).

### 2.4 Expression and purification of recombinant proteins

FhuD2 and Hla_H35L_ *S. aureus* proteins were purified as previously described (Irene et al., 2019). The genes encoding the *E. coli* BL21 proteins Agp, CpoB, EfeO, FepA, GlpQ, LamB, MalE, MalM, OppA, Pal, YdcL and YncD were amplified from chromosomal DNA and cloned as His‐TAG C‐terminal fusions using linearized pET15+ plasmid using PIPE method (Table S3). Proteins were purified using Ni‐NTA resin (Qiagen, Hilden, Germany) following standard purification procedures.

### 2.5 OMV purification and electrophoretic analysis

For assessment of OMV productivity of each mutant we used 50 ml culture volume, whereas OMVs for antigen expression analysis and batch preparation for immunization were purified from 200 ml and 1 L cultures, respectively. OMVs were collected from culture supernatants by filtration through a 0.22 μm pore size filter with a syringe for small volumes up to 50 ml (Merck, Darmstadt, Germany) or with a filter system for larger volumes (Starlab, Hamburg, Germany). Small volumes (50 ml and 200 ml) were applied to high‐speed centrifugation (174,900 x *g* for 2 h at 4°C) and pellets containing OMVs were resuspended in PBS (Gibco brand, Thermo Fisher Scientific, Waltham, MA USA). Purification of OMVs from cultures in bioreactor was carried out using Tangential Flow Filtration (ÄKTA flux system; GE Healthcare, Chicago, IL USA) with a Hollow Fibre cartridge UFP‐500‐C‐3MA (GE Healthcare, Chicago, IL USA). Protein content of OMVs was quantified using DC Protein Assay (Bio‐Rad) and the quality of OMVs was monitored by SDS‐PAGE loading OMVs (normalized by μg) on Any kD Criterion TGX Stain‐Free Protein Gel (Bio‐Rad Laboratories, Hercules, CA USA) stained using ProBlue Safe Stain (Giotto Biotech, Sesto Fiorentino, Italy). The detailed purification protocol has been submitted to EV‐TRACK (ID EV200158).

2‐DE was performed as previously reported (Fantappiè et al., 2017; Gagliardi et al., 2016). For analytical and MS‐preparative gels, 250 μg and 500 μg of protein, respectively, were diluted in 350 μL of denaturation buffer and 0.2% or 2% (v/v) IPG‐buffer (pH 3–10; GE Healthcare, Chicago, IL USA). Analytical gels and MS‐preparative gels were stained with ammoniacal silver nitrate and MS‐compatible silver staining, respectively, before being scanned using the ImageScanner III (GE Healthcare, Chicago, IL USA). Image analysis was performed on 2‐DE analytical gels using the ImageMaster 2D Platinum v.6.0 software (GE Healthcare, Chicago, IL USA). For each OMV preparation, OMVs*_ΔompA_* and OMVs*_Δ60_*, image analysis was carried out on three different spot maps. Qualitative differences between OMVs*_ΔompA_* and OMVs*_Δ60_* were considered significant only when absence/presence of protein spots was observed in all three corresponding gels.

### 2.6 Negative staining electron microscopy method

A volume of 5 μl of OMV diluted at 80 ng/μl in PBS were loaded onto a copper 200‐square mesh grid of carbon/formvar rendered hydrophilic by glow discharge using a Q150R S (Quorum, East Sussex, UK). The excess solution was blotted off after 30 s using Whatman filter Paper No.1. The grids were negatively stained with NanoW (Nanoprobes, Yaphank, NY USA) for 30 s, then blotted using Whatman filter Paper No.1 and finally let air dry. Micrographs were acquired using a Tecnai G2 Spirit Transmission Electron Microscope equipped with a CCD 2kx4k camera at a final magnification of 120000x.

### 2.7 Protein identification by mass spectrometry

Protein identification was carried out by peptide mass fingerprinting (PMF) using an Ultraflex III MALDI‐TOF/TOF mass spectrometer (Bruker Daltonics, Billerica, MA USA), equipped with a 200 Hz smartbeam I laser, as already described (Fantappiè et al., 2017; Gagliardi et al., 2016). Mass spectra were acquired in reflector positive mode with a laser frequency set to 100 Hz. Flex Analysis software v.3.0 was used for analysis. Acquired spectra were internally calibrated using auto‐proteolytic trypsin peptides (842.509 m/z and 2,211.105 m/z) and contaminants were removed from the obtained mass lists. PMF searching was carried out against entries for *E. coli* in the SwissProt database using Mascot software (Matrix Science Ltd., London, UK, www.matrixscience.com) with 100 ppm as peptide tolerance, monoisotopic peptide mass, allowing one missed cleavage by trypsin, carbamidomethylation of cysteine as fixed modification, and oxidation of methionine as variable modification. The parameter used to accept identifications was the default Mascot protein score greater than 56, corresponding to a 5% probability that the observed match was a random event (*P* < 0.05). The number of matched peptides and the extent of sequence coverage were also considered to confirm protein identification. Protein spots that emerged as significant qualitative differences along with the MS data for those that were successfully identified by MS are reported in Table S2.

### 2.8 Assessment of phospholipid content, particle size and dimension of OMVs

For lipid quantification, styrylic membrane dye FM4‐64 (Thermo Fisher Scientific, Waltham, MA USA) at a final concentration of 3.3 μg/ml was added to 125/250 ng of OMVs (protein content) resuspended in PBS in a black 96‐well OptiPlate (Perkin Elmer, Waltham, MA USA). As standard, we used *E. coli* L‐α‐phosphatidylglycerol (Merck, Darmstadt, Germany) in a range between 5 μg and 0.15 μg. The reaction was incubated for 10 min at 37°C and fluorescence (excitation at 485 nm, emission at 670 nm) was measured with an Infinite M200PRO plate reader (Tecan, Männedorf, Switzerland).

Assessment of particle size and dimension on OMVs diluted in PBS to a concentration of 100 ng/ml was performed using a Nanosight NS300 (Malvern Instruments, Malvern, UK) with a 532 nm laser, camera level 15, slider shutter and gain of 1206 and 245, respectively. Three videos of 60 s were recorded and analyzed with the software NTA 3.4 Build 3.4.003.

### 2.9 Toll‐Like‐Receptor agonistic activity and IL‐6 stimulation

OMV agonistic activity of human Toll Like Receptor 2 (hTLR2) and mouse Toll Like Receptor 4 (mTLR4) was measured using HEK‐Blue™ hTLR2 or HEK‐Blue™ mTLR4 Cells, respectively, and QUANTI‐Blue (InvivoGen, San Diego, CA USA) as substrate as previously described (Irene et al., 2019). For the IL‐6 assay, THP‐1 human leukemic monocyte cells were cultured in RPMI 10% FBS and differentiated into macrophages by adding 10 ng/ml of phorbol 12‐myristate 13‐acetate (PMA) and incubating cells at 37°C 5% CO_2_ for 48 h. Then 4 × 10^5^ cells in a volume of 10 μl/well of RPMI culture medium were aliquoted into a 96 well‐plates (Corning, Corning, NY USA). Different concentration of OMVs and purified LPS (control) were added to 90 μl/well of RPMI in duplicate starting from an initial concentration of 10,000 ng/ml to a final concentration of 10 pg/ml with 10‐fold serial dilutions. Cells and OMVs were incubated at 37°C O/N. IL‐6 release in supernatants was measured by Human IL‐6 Uncoated ELISA™ Kit (Thermo Fisher Scientific, Waltham, MA USA). Corning Costar ELISA plates were coated with 100 μL/well of anti‐human IL‐6 antibodies and incubated at 4°C O/N. The day after 100 μL/well of cell supernatants were transferred to the plate and incubated 2 h at room temperature (RT) and the assay was completed following the manufacturer's protocol. Plates were read at 450 nm using a SpectraMax M2 Microplate reader (Molecular Devices, San José, CA USA).

### 2.10 Animal experiments

All mice were purchased from Charles River Laboratories (Wilmington, MA USA). Animal experiments were carried out in accordance with experimental protocols reviewed and approved by the Animal Ethical Committees of University of Trento (Trento, Italy) and Toscana Life Sciences (Siena, Italy) and by the Italian Ministry of Health. Animal health was evaluated using a 0 (no signs of stress and suffering) to 6 (severe signs of stress and suffering) ‘pain score’ scale approved by the Animal Welfare Committees of University of Trento and Toscana Life Sciences in accordance with the ‘Working document on a severity assessment framework ‐ National Competent Authorities for the implementation of Directive 2010/63/EU on the protection of animals used for scientific purposes’. The evaluation considers the recommendations of the Federation of European Laboratory Animal Science Associations (FELASA) (Baumans et al., 1994). This ‘pain score’ scale is based on objective parameters and are assigned as follows: score 0: normal hair, mucosa, physical activity and nourishment; score 1: ruffled hair coat (piloerection), lesions or dehydration; score 2: as in 1 and reduced physical activity (e.g., grooming, exploration); score 3: as in 2 and lethargy and loose < 10% body weight; score 4: as in 3 and signs of respiratory impairment (dyspnoea or tachypnea); score 5: as in 4 and loss of body weight between 10% and 20%, lordosis or kyphosis; score 6: as in 5 and body weight loss > 20% (animals have to be sacrificed). For antibody titers, female BALB/c mice (5 per group) were immunized intraperitoneally (i.p.) on days 0, 14 and 28 with 2 μg of OMVs in the presence of Alum (2 mg/ml). Blood was collected by cardiac puncture from euthanized mice at day 35 and serum was obtained from blood through centrifugation at 2,000 rpm for 10 min. For T cell analysis, C57BL/6 mice were subcutaneously (s.c.) injected on days 0 and 7 with 10 μg of OMVs*_Δ_*_60_ + 5 μg of either OVA or SV40 synthetic peptides. At day 12, spleens were collected and used to perform intracellular cytokine staining for subsequent analysis.

### 2.11 Analysis of OMV immunogenicity by western blot and ELISA

Purified recombinant *E. coli* proteins (1 μg each) or total OMV proteins (10 μg) were loaded on Any kD Criterion TGX Stain‐Free Protein Gel (Bio‐Rad, Laboratories, Hercules, CA USA) and then transferred onto a nitrocellulose membrane using iBlot transfer stack (Thermo Fisher Scientific, Waltham, MA USA). Sera from mice immunized with OMVs*_ΔompA_* and OMVs*_Δ60_* were used at 1:1,000 dilutions and immunogenic proteins were detected with anti‐mouse IgG‐HRP antibodies (Agilent, Santa Clara, CA USA) at a dilution of 1:2,000 using ECL Select Western Blotting Detection Reagent (GE Healthcare, Chicago, IL USA).

Antigen specific antibody titers elicited by engineered OMVs were measured by ELISA as previously described (Irene et al., 2019) using FhuD2, Hla_H35L_, *Burkholderia pseudomallei* flagellin epitope (Bp) as recombinant proteins and D8‐FAT peptide (IQVEATDKDLGPNGHVTYSIVTDTD; Genscript Biotech, Piscataway Township, NJ USA) (200 ng each).

### 2.12 HLA neutralization assay

HLA neutralization assay was performed as previously described (Irene et al., 2019).

### 2.13 Analysis of OVA and SV40‐specific T cells

Percentage of OVA‐ and SV40‐specific T cells in immunized mice was performed as previously described (Grandi et al., 2018). Statistical significance was assessed using two‐tailed Student's *t*‐test.

## 3 RESULTS

### 3.1 Selection of endogenous proteins to be eliminated from *E. coli*‐derived OMVs

With the final goal to create an *E. coli* derivative releasing ‘proteome‐minimized’ OMVs, we first experimentally defined the proteome of OMVs from the hypervesiculating strain *E. coli* BL21(DE3)*ΔompA* by using two‐dimensional gel electrophoresis (2‐DE) coupled to mass spectrometry analysis (MS) as described in Fantappiè et al (Fantappiè et al., 2017). A total of 128 sequences were identified corresponding to 81 unique *E. coli* proteins. 2‐DE‐MS was coupled to bioinformatics analysis of the *E. coli* BL21(DE3) genome aimed at predicting all periplasmic and outer membrane proteins. We selected 504 proteins, which included all the 81 proteins identified by MS. Next, from these 504 proteins we excluded those ones that were classified as “indispensable” according to the Keio library (Baba et al., 2006), and we finally generated a priority list of 90 proteins, 63 of which identified by MS on 2D map, to be eliminated from our progenitor strain *E. coli* BL21(DE3)*ΔompA* (Table S1).

### 3.2 Construction of *E. coli* BL21(DE3)*Δ60* releasing proteome‐minimized OMVs

**FIGURE 1:**
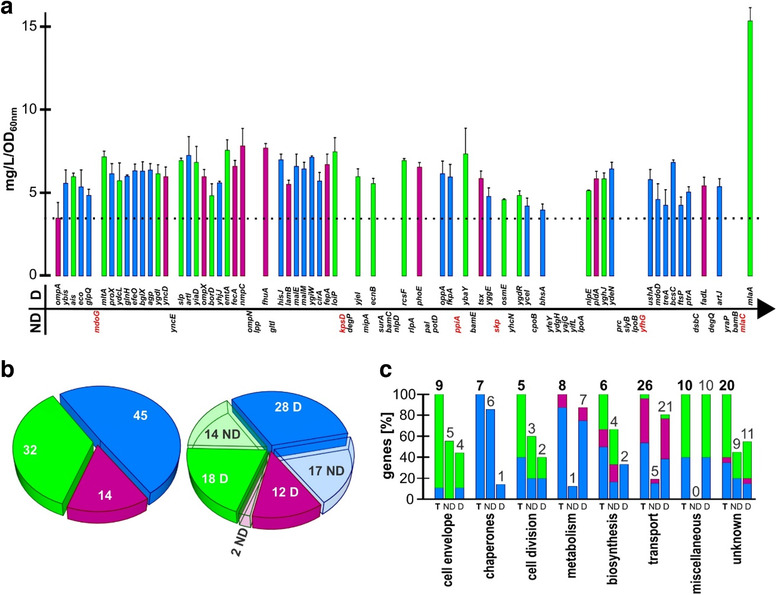
Creation of *E. coli* BL21(DE3)*Δ60* strain releasing proteome‐minimized OMVs. (a) The selected 90 genes (+ *ompA*) encoding dispensable periplasmic and outer membrane proteins are reported above and below the arrow according to the order used for CRISPR/Cas9‐guided inactivation. ‘D’ indicates the genes that were successfully inactivated and ‘ND’ those genes whose inactivation either failed or was obtained but resulted in an impaired growth and/or OMV production and therefore were excluded (written in ‘red’). For each mutant strain, the average (n = 3) OMV productivity (mg OMV (protein‐based)/litre/OD_600_ of culture) is reported and compared to the productivity of the progenitor *E. coli* BL21(DE3)*ΔompA* strain (dotted line). Bar colours indicate genes encoding outer membrane proteins (magenta), lipoproteins (green), periplasmic proteins (blue). Error bars, mean ± s.d. (b) *Left pie chart*. Classification of the 91 proteins (90 selected proteins + OmpA) according to their predicted compartmentalization (colour code as in a). The total number of proteins belonging to each category is written inside each slice. *Right pie chart*. Same representation as on the left, in which each slice is subdivided to highlight the number of the corresponding genes that were deleted (D, solid slices) or not‐deleted (ND, open slices). (c) The 90 selected genes + *ompA* are grouped into eight categories according to the biological/physiological function of their protein products. Bars (colour code as in A) indicate the total number of genes (T) and the number of deleted (D) and non‐deleted (ND) genes of each category

We previously published a CRISPR/Cas9‐based genome editing protocol, which allows the cumulative inactivation of any dispensable gene in *E. coli* at a pace of one mutation every two working days (Zerbini et al., 2017). By using this protocol, we attempted the stepwise inactivation of the 90 selected genes by deleting 30 nucleotides at the 5′ end of the coding region of each mature protein and by introducing an in‐frame stop codon (Figure 1a, Fig. S1). Before moving to the next gene editing cycle, growth kinetics and OMV productivity was assessed to exclude mutants with impaired growth and/or OMV productivity. At the end of this *brute force* approach, a mutant, named *E. coli* BL21(DE3)*Δ58*, was generated, which carries, in addition to the mutation in the *ompA* gene present in the progenitor *E. coli* BL21(DE3)*ΔompA* strain, the inactivation of 57 out of 90 genes (63%). Of the remaining 33 genes, six could be deleted in the presence of the preceding mutations (written in red in Figure 1a) but the mutants had impaired growth kinetics, while the inactivation of the other 27 genes was not compatible with the genetic background of the recipient strains. Most of the non‐viable mutations were lipoproteins and periplasmic proteins (Figure 1b) and included genes involved in cell envelope assembly (5 out of 9) and molecular chaperones activity (6 out of 7) (Figure 1c). Interestingly, all mutants showed an OMV productivity higher than the *E. coli* BL21(DE3)*ΔompA* progenitor, with some mutations (*mltA*, *bglX*, *emtA* and *mlaA*) appearing particularly effective in promoting vesiculation (Figure 1a). Finally, to reduce the potential reactogenicity of LPS‐associated OMVs, the genome editing process was completed with the addition of *msbB* and *pagP* gene inactivation, which results in the synthesis of a penta‐acylated Lipid A (Somerville et al., 1996; Vorachek‐Warren et al., 2002). The final strain, carrying a total of 60 gene deletions, was named *E. coli* BL21(DE3)*Δ*60.

**FIGURE 2:**
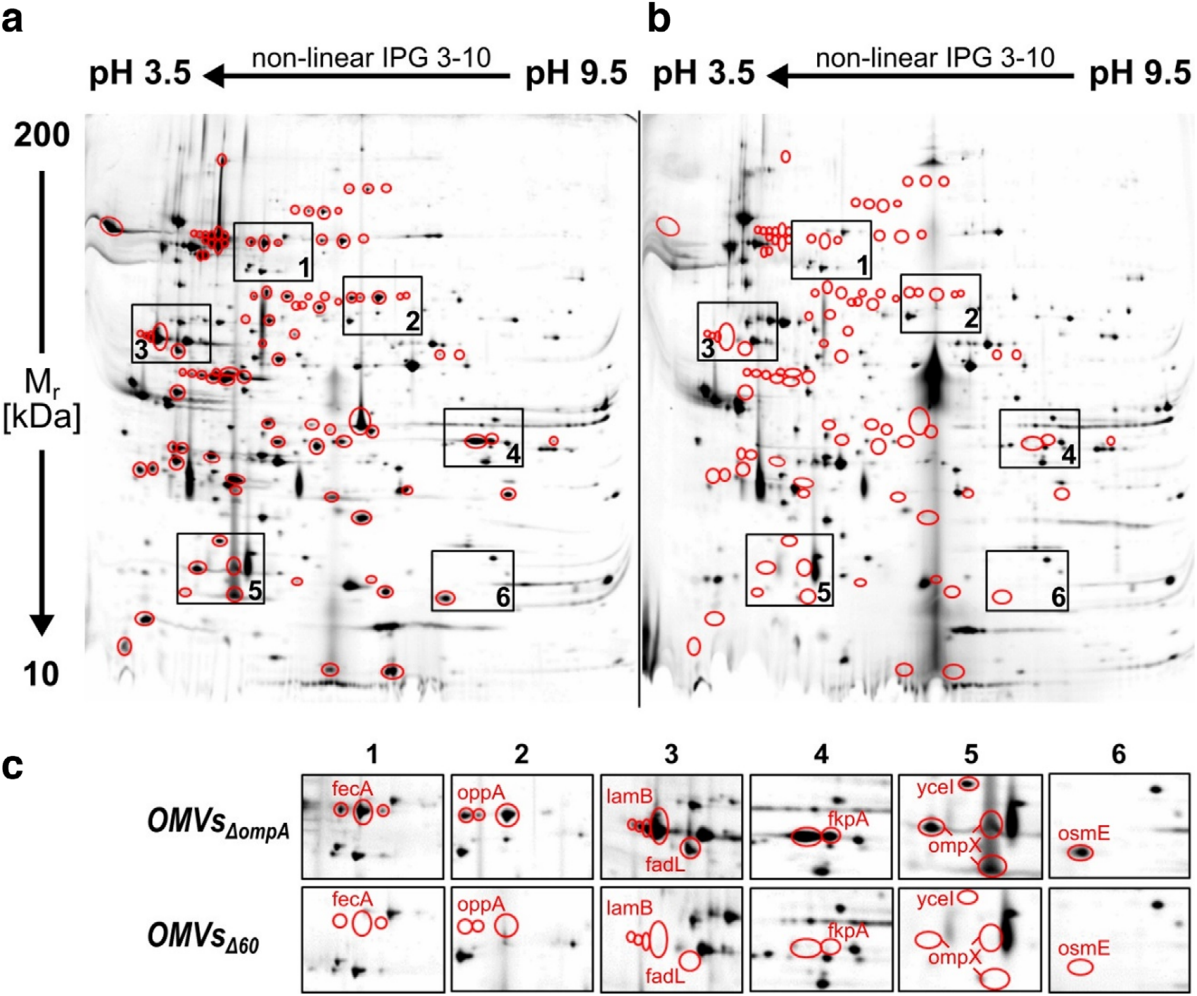
Comparison of two‐dimensional electrophoresis of OMVs*_ΔompA_* and OMVs*_Δ60_* ‐ Silver stained 2‐DE gels of OMVs from *E. coli* BL21(DE3)*ΔompA* (a) and *E. coli* BL21(DE3)*Δ60* (b). Red circles visualize those protein spots visible in OMVs*_ΔompA_* and not visible in OMVs*_Δ60_*. All spots not present in OMVs*_Δ60_* were removed from the OMVs*_ΔompA_* gel, digested with trypsin and analyzed by mass spectrometry. Zoom areas (c) are taken as examples of qualitative differences between the two gels and report the ID of the MS‐identified proteins (see Table S1 for annotations)

The correctness of each gene inactivation was first verified by PCR and sequence analyses using primers spanning the mutated regions (Fig. S1) and subsequently by sequencing of the whole genome that has been deposited at GenBank under the accession JADXDS000000000. Moreover, the disappearance from the vesicular compartment of the proteins encoded by the deleted genes was followed by comparing the 2D map of OMVs*_Δ60_* and OMVs*_ΔompA_*. A total of 98 protein spots were no longer visible in the 2‐DE map of OMVs*_Δ60_* (Figure 2). Of these 98 spots, 75 were successfully identified in OMVs*_ΔompA_* 2‐DE map by MS analysis, and they corresponded to 46 unique proteins, 43 of which encoded by the deleted genes (Table S2 and Fig. S2). The disappearance of three proteins not encoded by the deleted genes (Table S1) could be ascribed to the fact that their expression level, which was already low in OMVs*_ΔompA_*, could be further reduced by the inactivation of other genes. As far as the remaining 16 out of the 59 inactivated proteins are concerned, two are MsbB and PagP and the other 14 belong to the group of proteins that were selected by bioinformatics analysis. From the 2D map analysis of OMVs*_Δ60_* the disappearance of the 59 proteins did not correspond to a substantial increase of specific protein spots. The stained material that appeared at the centre of the 2D gel probably represents a technical artefact since it does not carry proteinaceous material, as judged by mass spectrometry analysis.

### 3.3 Properties of *E. coli* BL21(DE3)*Δ60* and OMVs*_Δ60_*

**FIGURE 3:**
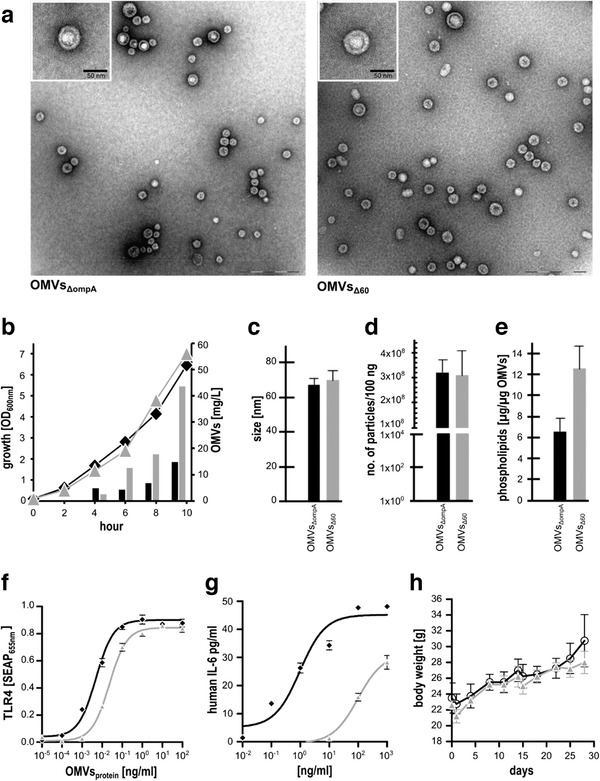
Properties of *E. coli* BL21(DE3)*Δ60* and OMVs*_Δ_*_60_. (a) *Morphology of OMVs_ΔompA_ and OMVs_Δ60_ analyzed by electron microscopy*. (b) *Comparison of growth kinetics and OMV productivity between E. coli BL21(DE3)Δ60 and E. coli BL21(DE3)ΔompA. E. coli* BL21(DE3)*Δ60* (▲) and *E. coli* BL21(DE3)*ΔompA* (♦) were grown in a bioreactor over a 10 h period and 50 ml cultures were collected at different time points and used for OMV purification and quantitation. OMVs productivity is given as mg/L (protein content). (c) *Determination of OMV size ‐* Purified OMVs were characterized using a Nanosight NS300. The size value is given as an average of two independent biological replicates, analyzed in duplicate. (d) *Determination of particle number ‐* OMVs were analyzed at a normalized concentration of 100 ng/ml and the number, determined using Nanosight NS300, represents the average value obtained from two OMV preparations and two independent analyses. (e) *Determination of lipid content in OMVs ‐* Styrylic membrane dye FM4‐64 (Thermo Fisher Scientific, Waltham, MA USA) at a final concentration of 3.3 μg/ml was added to 125 and 250 ng of OMVs. After 10 min at 37°C fluorescence was measured (excitation at 485 nm, emission at 670 nm) and lipid content was estimated as μg/μg OMVs (protein based) using *E. coli* L‐α‐phosphatidylglycerol as standard. (f) *Analysis of TLR4 agonistic activity of OMVs ‐* HEK‐Blue mTLR4 cells were incubated with different amounts of either OMVs*_ΔompA_* (♦) or OMVs*_Δ60_* (▲). After 17 h the levels of secreted alkaline phosphatase (SEAP) secreted upon mTLR4 stimulation by OMVs were determined by reading the absorbance of the culture supernatant at 655 nm in duplicate. (g) *In vitro release of IL‐6 by macrophages upon OMV stimulation* ‐ THP‐1 human leukemic monocyte cells (4 × 10^5^ cells in 10 μL/well) differentiated to macrophages by PMA were incubated with different concentrations of OMVs*_ΔompA_* (♦) and OMVs*_Δ60_* (▲) in a final volume of 100 μL/well. IL‐6 release in the supernatants was measured by Human IL‐6 Uncoated ELISA Kit (Thermo Fisher Scientific, Waltham, MA USA) in duplicate. (h) *Body weight analysis of mice immunized with OMVs_Δ60_* ‐ Mice (5 mice/group) were immunized three times at 2‐week intervals with 10 μg of OMVs*_Δ_*_60_/dose in 100 μL PBS containing 2 mg/ml of Alum hydroxide. Immunization with 100 μL PBS containing 2 mg/ml of Alum hydroxide was used as control. Body weight of each mouse was measured at 3–5 day intervals over a period of 30 days. Curves are reported as average body weight (n = 5) of mice immunized with OMVs*_Δ_*_60_ (▲) or alum (○). Error bars in C‐F and H: mean ± s.d. Error bars in F and G: mean ± s.e.m.

Compared to its progenitor *E. coli* BL21(DE3)*ΔompA*, *E. coli* BL21(DE3)*Δ60* had similar growth kinetics, but released approximately 3‐folds more OMVs (Figure 3b). OMVs from both strains were purified from the culture supernatants and their size and quality was analyzed by electron microscopy and light scattering using a Nanosight NS300 set with 532 nm laser. As shown in Figure 3a, the size and the morphology of the vesicles from both samples looked very similar by electron microscopy, with no visible contamination or presence of aggregates. The similarity in size and number of vesicles/μg of total proteins of OMVs*_ΔompA_* and OMVs*_Δ60_* was confirmed by Nanosight analysis (Figure 3c, d), while the elimination of endogenous proteins (and probably the modification of the *mla* pathway) affected the phospholipid content, which was approximately twice as much in OMVs*_Δ60_* with respect to OMVs*_ΔompA_* (Figure 3e). In line with the inactivation of *msbB* and *pagP* genes, OMVs*_Δ60_* had a TLR4 agonistic activity and IL‐6 stimulation capacity approximately one and three orders of magnitude lower than OMVs*_ΔompA_*, respectively (Figure 3f, g). These values appear to be in line with what reported for other OMV preparations for human use (Gerke et al., 2015; Ladhani et al., 2016; Rossi et al., 2016) and the safety profile of OMVs*_Δ60_* was further confirmed by the absence of body weight loss in immunized mice (Figure 3h). Indeed, all OMVs*_Δ60_* immunized mice experienced a ‘pain score’ 0/1, showing regular or slightly ruffled hair coat, healthy mucous membranes, normal physical activity and normal consumption of food and water. Considering that the 59 inactivated proteins include a number of lipoproteins, we also compared the TLR2 agonistic activity of OMVs*_Δ60_* and OMVs*_ΔompA_*. However, only a non‐statistically significant decrease of TLR2 activation by OMVs*_Δ60_* was observed (Fig. S3), suggesting that, in addition to lipoproteins, other OMV components can stimulate this innate immunity receptor.

**FIGURE 4:**
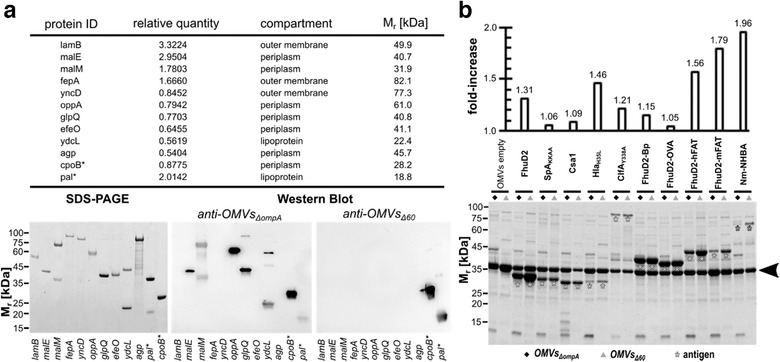
Immunogenicity of endogenous OMV proteins and expression of heterologous antigens in OMVs ‐ (a) Ten proteins identified in OMVs*_Δ_*_ompA_ by mass spectrometry were expressed as His‐TAG fusions in *E. coli* BL21(DE3) and purified by IMAC. Purified proteins were analyzed by SDS‐PAGE and Western Blot using sera from mice immunized with either OMVs*_Δ_*_ompA_ or OMVs*_Δ_*_60_. As a control, two proteins (*) present in both OMVs***_Δ_***_ompA_ and OMVs*_Δ_*_60_ were also analyzed. Relative quantity refers to the number of pixel of the corresponding protein spot(s) in relation to the sum of pixel from all protein spots (= 100). (b) ‐ *E. coli* BL21(DE3)*ΔompA* and *E. coli* BL21(DE3)*Δ60* strains expressing different heterologous antigens as lipoproteins were grown in LB at 30°C. At OD_600_ = 0.5, 0.1 mM IPTG was added and after 2 h OMVs were purified from culture supernatants by ultracentrifugation. Aliquots corresponding to 7.5 μg of total OMV proteins were loaded on each lane. The amount of recombinant antigens expressed in the OMVs was estimated by densitometry analysis of each lane using the formula: *μg recombinant protein = μg OMVs x (pixels recombinant protein)/(total lane pixels)*. The upper panel indicates the ratio between the amount of each recombinant protein expressed in OMVs*_Δ_*_60_ and the amount of the same protein expressed in OMVs*_Δ_*_ompA_ (see also Table S7). The arrowhead indicates the band corresponding to the most abundant protein (OmpF) in empty OMVs

One of the main motivations to create a strain releasing proteome‐minimized OMVs was to reduce the immune responses toward OMV endogenous proteins and to enhance the immunogenicity of heterologous antigens. To test this, two proteins (the products of *cpoB* and *pal* genes) present in both OMVs*_Δ60_* and OMVs*_ΔompA_* and ten proteins only present in OMVs*_ΔompA_*, were expressed in *E. coli*, purified and analyzed by Western Blot using sera from mice immunized with either OMVs*_Δ60_* or OMVs*_ΔompA_*. While CpoB and Pal proteins were stained by both sera, five out of the ten proteins absent in OMVs*_Δ60_* were recognized only by the sera from OMVs*_ΔompA_*‐immunized mice (Figure 4a). Therefore, as expected, several OMVs endogenous proteins are immunogenic and their removal silenced the corresponding specific immune responses. To investigate whether the absence of some of OMV proteins could enhance the immune response toward the others, we separated the total proteins of OMVs*_ΔompA_* and OMVs*_Δ60_* on SDS‐PAGE and we analyzed them by Western Blot using sera from mice immunized with either OMVs*_ΔompA_* or OMVs*_Δ60_*. As shown in Fig. S4, the anti‐OMVs*_ΔompA_* serum recognized a higher number of protein species in OMVs*_ΔompA_* than in OMVs*_Δ60_*. Moreover, no additional reactive proteins were observed in both OMVs*_ΔompA_* and OMVs*_Δ60_* using sera from mice immunized with OMVs*_Δ60_*. Therefore, it appears that the poorly immunogenic proteins continue to remain so even if their relative abundance in OMVs would partially change as a result of the inactivation of others.

Finally, we asked the question whether the inactivation of endogenous proteins could affect the stability of OMVs. To test this we incubated the OMV preparations at different temperatures and we followed their stability over a period of three months using SDS‐PAGE. No appreciable difference in the protein pattern could be detected even if OMVs were conserved at 37°C.

### 3.4 Engineering of OMVs*_Δ60_* with heterologous proteins

We next investigated whether the removal of the OMV endogenous proteins could have affected the loading capacity of OMVs. We previously showed that foreign antigens and epitopes efficiently compartmentalize in OMVs when their coding sequences are either directly fused to a lipoprotein leader sequence (Irene et al., 2019) or hooked to the C‐terminus of an OMV‐associated lipoprotein (Grandi et al., 2017, 2018). Therefore, we selected ten heterologous proteins and epitopes, we expressed them as lipidated antigens in both *E. coli* BL21(DE3)*ΔompA* and *E. coli* BL21(DE3)*Δ60* and we purified the vesicles from each recombinant strain. Purified vesicles were analyzed by SDS‐PAGE and the amount of recombinant protein in each vesicle preparation was determined by densitometry analysis (Figure 4b). From these results, two main conclusions can be drawn. First, and in line with recently reported data (Irene et al., 2019), heterologous proteins efficiently compartmentalized in OMVs, where they could account for as much as 20%–30% of total OMVs proteins (Table S7). Second, the loading capacity of heterologous proteins was higher in OMVs*_Δ60_* than OMVs*_ΔompA_*, with an increase that could be as high as 90% (Figure 4b).

Finally, we asked the question whether the expression of heterologous antigens could have affected the stability of OMVs. To test this, we analyzed the OMV protein pattern after storing FhuD2‐hFAT1‐OMVs*_ΔompA_* and of FhuD2‐hFAT1‐OMVs*_Δ58_* at different temperatures (OMVs*_Δ58_* derived from *E. coli* BL21(DE3)*Δ58*, a strain which differs from to *E. coli* BL21(DE3)*Δ60* only for the absence of the *msbB* and *pagP* mutations. OMVs*_Δ58_* have an identical proteome as OMVs*_Δ60_*). Similarly to what observed with OMVs*_Δ60_* and OMVs*_ΔompA_*, the stability of the engineered OMVs was not impaired even after three‐month incubation at 37°C.

### 3.5 OMVs*_Δ60_* as vaccine platform

**FIGURE 5:**
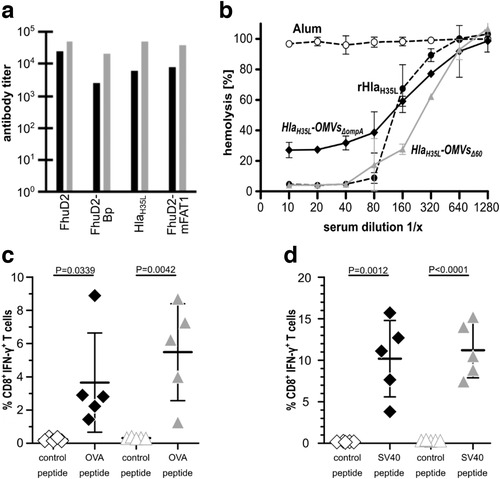
Immunogenicity and functional immune responses in mice immunized with OMVs*_Δ_*_60_ decorated with heterologous antigens/epitopes – (a) *IgG titers in mice immunized with OMVs decorated with lipidated heterologous antigens*. Groups of five female BALB/c mice were immunized i.p. 3 times at two‐week intervals with 2 μg of engineered OMVs*_Δ_*_ompA_ (black bars) and OMVs*_Δ_*_60_ (grey bars) in Alum. Sera were collected 7 days after the third immunization and IgG titers were measured in triplicate by ELISA, coating the plates (200 ng/well) with either the corresponding purified recombinant antigen (FhuD2 and Hla_H35L_) or the corresponding synthetic peptide (Bp and mFAT1). (b) *Functional activity of anti‐HLA antibodies elicit by Hla_H35L_‐OMVs_Δ60_* – Purified Hla was incubated with rabbit erythrocytes in the presence or absence of different dilutions of sera from mice immunized with Hla_H35L_‐OMVs*_Δ_*_60_, Hla_H35L_‐OMVs*_Δ_*_ompA_, or purified recombinant Hla_H35L_ (10 μg) in Alum. Inhibition of Hla hemolytic activity is given as percentage of hemolytic activity obtained incubating the rabbit erythrocytes with water. Error bars: mean ± s.d. (c and d) *Analysis of epitope‐specific CD8^+^ T cells elicited by immunization with OMVs absorbed with synthetic peptides corresponding to OVA and SV40 CD8^+^ T cell epitopes* ‐ C57BL/6 mice (5 animals/group) were immunized with 10 μg of OMVs*_Δ_*_ompA_ or OMVs*_Δ_*_60_ ‘absorbed’ to 5 μg of either OVA peptide or SV40 peptide. After 5 days, splenocytes were collected from each animal and peptide‐specific IFN‐γ producing CD8^+^ T cells were counted by flow cytometry, after stimulation with the specific peptide (OMVs*_ΔompA_* ♦ and OMVs*_Δ60_* ▲) or an irrelevant peptide (open symbols). Error bars: mean ± s.e.m.

To test whether OMVs*_Δ60_* decorated with heterologous antigens/epitopes could elicit antigen/epitope‐specific immune responses, we selected four engineered OMVs*_Δ60_* loaded with lipidated FhuD2, Hla_H35L_, FhuD2‐mFAT1 fusion and FhuD2‐Bp fusion and we used them to immunize (i.p.) groups of BALB/c mice. FhuD2, Hla_H35L_ are two *S. aureus* protective antigens (Irene et al., 2019). FhuD2‐mFAT1 is a C‐terminal fusion carrying the murine homolog of a surface‐exposed epitope of FAT1, a protein found overexpressed in colorectal cancer (Grandi et al., 2018; Pileri et al., 2016). Finally, FhuD2‐Bp is a fusion carrying three copies of an immunogenic epitope (TRMQTQINGLNQGVSNAND) from *Burkholderia pseudomallei* flagellin separated by a short linker sequence (GS). After immunization, sera from animals of each group were pooled and antigen‐specific antibody titers were measured by ELISA. High IgG titers were induced against all engineered antigens and in the case of the OMVs*_Δ_*_60_ decorated with Hla_H35L_, FhuD2‐mFAT1 and FhuD2‐Bp the titers were approximately five to ten‐fold higher than what obtained with animals immunized with engineered OMVs deriving from the progenitor *E. coli* BL21(DE3)*ΔompA* strain (Figure 5a). The higher immunogenicity of engineered OMVs*_Δ60_* was also confirmed by analyzing the ELISA titers of antigen‐specific antibodies from single mouse sera. As shown in Fig.S6, sera from OMVs*_Δ60_*–immunized mice had consistently higher levels of antigen‐specific antibodies with respect to OMVs*_ΔompA_*–immunized mice. Finally, in the case of Hla_H35L_, we also determined the inhibition of Hla haemolytic activity of sera from mice immunized with engineered OMVs. Sera from mice immunized with Hla_H35L_‐OMVs*_Δ60_* inhibited haemolysis at a three‐fold higher dilution with respect to sera from Hla_H35L_‐OMVs*_ΔompA_* ‐immunized mice (Figure 5b). Moreover, the anti‐Hla_H35L_‐OMVs*_ΔompA_* sera were not able to inhibit completely the Hla hemolytic activity, suggesting that the two sera differ in both quantitative and qualitative terms.

To be broadly applicable, OMVs should elicit not only humoral immunity but also cell‐mediated immunity. Therefore, we tested the capacity of OMVs*_Δ60_* to induce cytotoxic CD8^+^ T cell responses. To this aim, 5 μg of synthetic peptides corresponding to OVA and SV40 epitopes were absorbed to 10 μg of both OMVs*_ΔompA_* and OMVs*_Δ60_* and the OMV‐peptide mixtures were used to immunize C57BL/6 mice. Animals were given two doses of vaccines, one week apart, and five days after the second immunization splenocytes were stimulated with the corresponding peptide and the frequency of IFN‐γ‐producing CD8^+^ T cells was determined by flow cytometry. Both immunizations elicited epitope‐specific T cells at a frequency ranging from 3% to 15% of total splenocytic CD8^+^ T cells (Figure 5c, d). In particular, OMVs*_Δ60_* had a tendency to elicit higher frequencies of T cells even though, for a rigorous statistical analysis, a higher number of animals should be used.

## 4 DISCUSSION

In this work, we have described the creation of an *E. coli* derivative, *E. coli* BL21(DE3)*Δ60*, in which 60 genes have been inactivated using a highly efficient CRISPR/Cas9‐based genome editing approach. The strain features the release of OMVs deprived of 59 endogenous proteins and carrying a penta‐acylated lipopolysaccharide. We also describe the main properties of the OMVs released by *E. coli* BL21(DE3)*Δ60* and how the strain can be exploited for the development of effective OMV‐based vaccines.

We believe that this work delivers a number of messages that can be of interest from technological, scientific and translational standpoints.

From a technical standpoint, we validated our CRISPR/Cas9‐based gene editing approach (Zerbini et al., 2017), which demonstrated that sequential, multiple gene manipulations could be accumulated in the same strain at a pace of one mutation every other day. With the present work, once the 90 genes were selected, it took us approximately one month to produce the corresponding 90 pCRISPR‐SacB‐gDNA plasmids and double stranded donor DNAs, and we completed the whole process of sequential gene knockouts in roughly 1 year (approximately two mutations/working week). Considering that our protocol allows the efficient simultaneous introduction of at least two mutations at a time, strains carrying selective gene modifications could theoretically be accelerated to a pace of four mutations/week.

**FIGURE 6:**
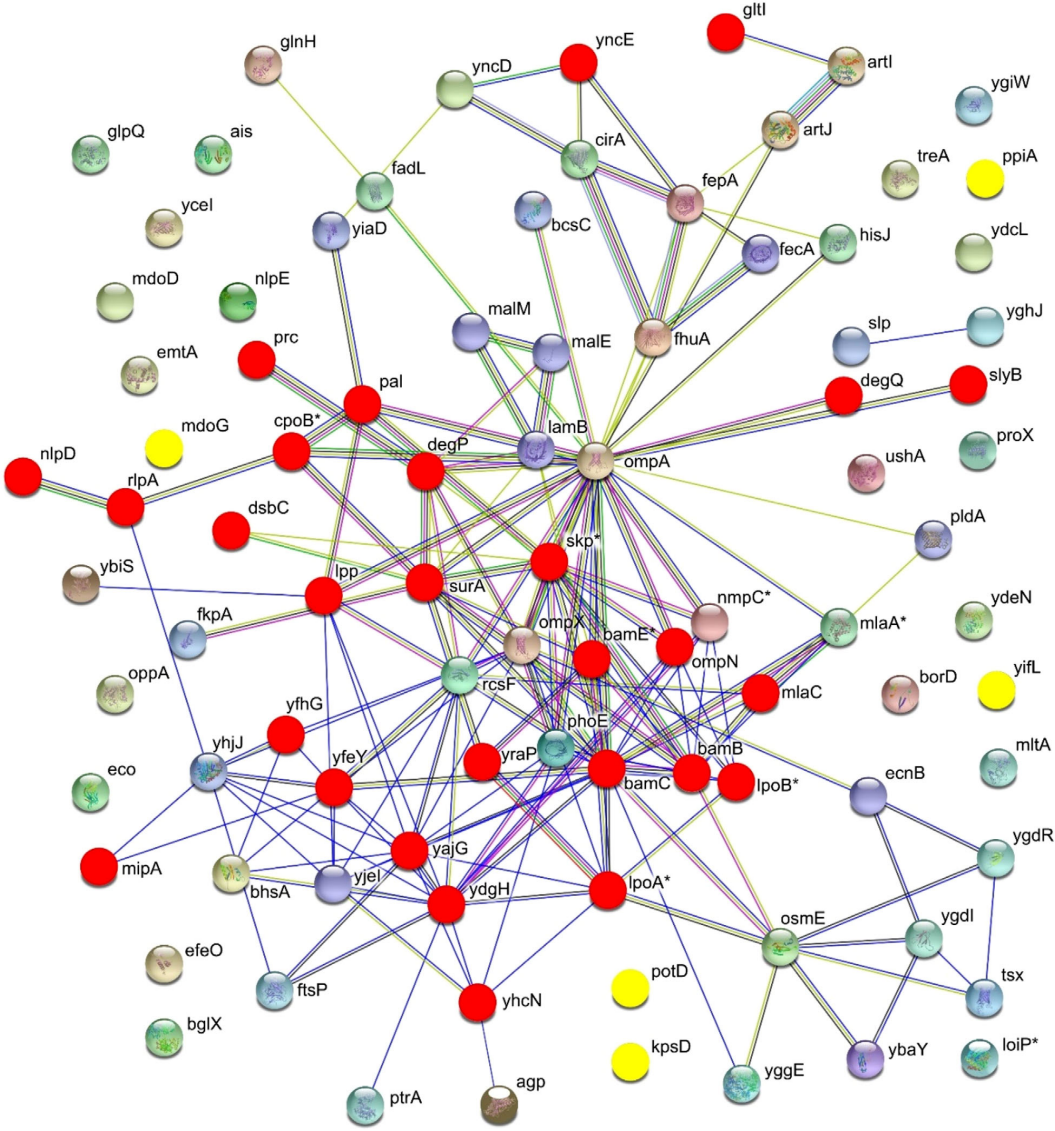
Physical and functional network among the 90 genes selected for gene knockout ‐ The 90 genes selected for gene knockout (Figure 1) were analyzed together with *ompA* using STRING database to visualize their possible physical (i.e. transcriptional) and functional association. Lines indicate which genes the database considers correlated according to different criteria and the number of connecting lines correlates with the number of association criteria. Red and yellow dots represent the genes whose inactivation failed or resulted in impaired growth and/or OMV productivity (see Discussion for more explanation). Asterisks indicate those proteins for which the STRING database has assigned different synonymous names with respect to those used in Figure 1 (see Table S1)

From a scientific standpoint, we believe that our work offers a collection of data, which might be useful to better understand the role of specific gene products and the complex network of protein‐protein interactions occurring in the periplasmic and outer membrane compartments. As stated above, all genes selected for this study were classified as ‘dispensable’ (Baba et al., 2006) and could be singularly inactivated without affecting viability (Zerbini et al., 2017). The fact that some combinations of gene deletions appeared to be not compatible (or at least were not selected using the screening procedure adopted in our protocol) opens the question as to which relationship these genes have with the others to make their deletion incompatible with survival under the growth conditions used. Addressing this question could be of particular interest for those genes whose encoded proteins are still classified as ‘hypothetical and/or unknown’. If we look at the network connecting all selected 90 genes as it emerges from STRING database (Szklarczyk et al., 2019), it appears that most of the ‘non‐permissive’ mutations fall in those genes/gene products that have some physical (i.e. transcriptional) or functional association with others (‘red nodes’ in Figure 6). One possible explanation is that at least some of the genes exert important redundant functions. For instance, *yncE*, a gene with unknown function, is connected with a number of genes (*fhuA*, *fepA*, *cirA*, *yncD*, *fecA*) all involved in iron metabolism/transport. Therefore, the simultaneous deletion of all six genes could impair iron assimilation to a non‐permissible level. Interestingly, five of the non‐permissive mutations, *mdoG*, *potD*, *kpsD*, *yifL* and *ppiA*, include genes that seem to have no relationship with any others (‘yellow dots’ in Figure 6). Such observation deserves further investigation.

Another interesting result from our study is that the removal of proteins did not substantially affect size and number of OMVs, as estimated after normalization to protein content (Figure 3c, d) and by electron microscopy analysis (Figure 3a). This could be explained assuming that OMVs carry a constant protein cargo and the loss of one protein species tends to be compensated with the accumulation of some others. This effect can be appreciated in Figure 4b: the intensity of the abundant OmpF band is higher in OMVs*_Δ60_* than OMVs*_ΔompA_* (lanes 1 and 2) and the accumulation of heterologous proteins parallels the reduction of other bands, predominantly OmpF. While size and number of vesicles/μg of protein remain unchanged, phospholipid content is higher in OMVs*_Δ60_* (Figure 3e). This difference could be due to the structural modification of Lipid A between OMVs*_Δ60_* and OMVs*_ΔompA_*. The absence of an acyl moiety might either affect the phospholipid determination or be compensated by the additional transport of phospholipids in the external leaflet of the outer membrane. Moreover, phospholipid accumulation is physiologically regulated by an ABC transport system, comprising a number of genes involved in the *m*aintenance of OM *l*ipid *a*symmetry (*mla* pathway) (Malinverni & Silhavy, 2009). It is likely that the inactivation of *mlaA* in *E. coli* BL21(DE3)*Δ60* contributes to the increase of phospholipid content in OMVs*_Δ60_* and to the hyper‐vesiculating phenotype, similarly to what observed in *Haemophilus influenzae, Vibrio cholerae* and *E. coli* (Kulp et al., 2015; Roier et al., 2016).

From a translational standpoint, we believe that *E. coli* BL21(DE3)*Δ60* can find concrete applications in vaccine development. As already pointed out, OMVs are becoming an attractive vaccine platform, but require optimization for a broad application. This study represents an important step forward in this regard. Essentially, *E. coli* BL21(DE3)*Δ60* releases vesicles with several important properties. First, the strain produces > 40 mg/L of vesicles (protein based) under the laboratory conditions we used. Such yield, which can surely be further optimized in large‐scale production processes, is nonetheless sufficient to guarantee the formulation of approximately 1,000 vaccine doses/L, assuming a 25–50 μg OMVs/dose for human applications. Second, we have shown that the elimination of 59 OMV endogenous proteins has a beneficial effect on the level of expression and immunogenicity of heterologous antigens. Recombinant proteins can be incorporated into OMVs at a level consistently higher (up to 90%) compared to the progenitor strain. Moreover, in most of the cases we analysed, antigen‐specific antibody titres elicited by engineered OMVs_Δ60_ were superior to what obtained with OMVs_ΔompA_. This could be a consequence of the higher antigen expression level or of the absence of the 59 endogenous proteins, which could otherwise interfere with, or dilute the antigen‐specific immune responses. Indeed, we showed that many endogenous proteins are immunogenic in the mouse model and therefore their removal should be beneficial for an optimal immune response against heterologous antigens, as demonstrated for a number of antigen, including Hla_H35L_ (Figure 5a, b).

Immune responses elicited by OMVs*_Δ60_* are not restricted to antibodies. Our data show that when formulated with two CD8^+^ T cell synthetic epitopes elevated frequencies of epitope‐specific CD8^+^ T cells were induced in immunized mice, with frequencies ranging from 3% to 8% and 7% to 15% in the case of OVA and SV40 epitopes, respectively. In our hands, other adjuvants already used in humans, such as CpG and Hiltonol, did not promote such elevated epitope‐specific CD8^+^ T cell frequencies. Considering the importance of T cell responses in cancer immunotherapy, our data suggest that OMVs*_Δ60_* can be exploited not only as ingredient for infectious disease vaccines but also for cancer vaccines.

Moreover, we showed that OMVs*_Δ60_* have a safety profile similar if not superior to what reported for the OMV vaccines already in clinical use (Gerke et al., 2015; Rossi et al., 2016), an important feature in view of their future human applications.

Finally, the OMV stability appears to be particularly striking. Although stability studies using different OMV preparations are still in progress, prolonged conservation of our vesicles even at room temperature does not modify their overall protein profile, and this is true for both OMVs*_ΔompA_* and OMVs*_Δ60_* as well as for the engineered OMVs analyzed so far. We are collecting 1‐year stability data on different OMV preparations and the results so far available confirm the robustness of the preparations. High stability is a remarkable property of our OMVs also in view of their application in developing countries where it would be difficult to guarantee a cold chain for temperature‐sensitive formulations.

Taking into account all advantageous characteristics presented here, we believe that *E. coli* BL21(DE3)Δ60 has the potential to become a workhorse factory for novel OMV‐based vaccines.

## AUTHOR CONTRIBUTIONS

Guido Grandi conceived and designed the study, analyzed data and wrote the manuscript; Yaqoub Ashhab coordinated the bioinformatics analysis; Ilaria Zanella, Enrico König and Francesca Zerbini performed knockouts; Carmela Irene, Michele Tomasi, Riccardo Corbellari, Alberto Grandi: OMV engineering; Ilaria Zanella, Enrico König, Michele Tomasi, Luca Frattini, Laura Fantappiè, Carmela Irene, Riccardo Corbellari, Samine J. Isaac, Martina Grigolato, and Alberto Grandi: OMV production and purification; Enrico König, Luca Frattini, Michele Tomasi, Assunta Gagliardi and Alberto Grandi: biochemical and biophysical characterization of OMVs; Michele Tomasi, Ilaria Zanella, Enrico König and Alberto Grandi: OMV immunogenicity; Michele Tomasi, Laura Fantappiè, Silvia Valensin, Samine J. Isaac and Martina Grigolato: mouse models; Ilaria Zanella, Assunta Gagliardi and Elena Caproni: *in vitro* assays; Enrico König and Carmela Irene: recombinant protein purification; Assunta Gagliardi and Luca Bini:2‐DE and MS analysis; Ilaria Ferlenghi and Fabiola Giusti electron microscopy analysis; Enrico König edited manuscript, Tables and Figures and all authors read, corrected and approved the final manuscript.

## CONFLICTS OF INTEREST

Guido Grandi, Alberto Grandi, Enrico König, Ilaria Zanella, Laura Fantappiè and Carmela Irene are co‐inventors of patents on OMVs; Alberto Grandi and Guido Grandi are involved in a biotech company interested in exploiting the OMV platform.

## Supporting information

Supplementary information

Supplementary information

## References

[jev212066-bib-0001] Altschul, S. F. , Gish, W. , Miller, W. , Myers, E. W. , & Lipman, D. J. (1990). Basic local alignment search tool. Journal of Molecular Biology 215, 403–410 2231712 10.1016/S0022-2836(05)80360-2

[jev212066-bib-0002] Baba, T. , Ara, T. , Hasegawa, M. , Takai, Y. , Okumura, Y. , Baba, M. , Datsenko, K. A. , Tomita, M. , Wanner, B. L. , & Mori, H. (2006). Construction of Escherichia coli K‐12 in‐frame, single‐gene knockout mutants: The Keio collection. Molecular Systems Biology 2, 1–11 10.1038/msb4100050 PMC1681482 16738554

[jev212066-bib-0003] Baumans, V. , Brain, P. F. , Brugére, H. , Clausing, P. , Jeneskog, T. , & Perretta, G. (1994). Pain and distress in laboratory rodents and lagomorphs. Laboratory Animals 28, 97–112 8035572 10.1258/002367794780745308

[jev212066-bib-0004] Berlanda Scorza, F. , Colucci, A. M. , Maggiore, L. , Sanzone, S. , Rossi, O. , Ferlenghi, I. , Pesce, I. , Caboni, M. , Norais, N. , Di Cioccio, V. , Saul, A. , & Gerke, C. (2012). High yield production process for Shigella outer membrane particles. PLoS One 7, e35616. 22701551 10.1371/journal.pone.0035616 PMC3368891

[jev212066-bib-0005] Bishop, D. G. , & Work, E. (1965). An extracellular glycolipid produced by Escherichia coli grown under lysine‐limiting conditions. The Biochemical Journal 96, 567–576 4953781 10.1042/bj0960567 PMC1207076

[jev212066-bib-0006] Chatterjee, S. N. , & Das, J. (1967). Electron microscopic observations on the excretion of cell‐wall material by Vibrio cholerae. Journal of General Microbiology 49, 1–11 4168882 10.1099/00221287-49-1-1

[jev212066-bib-0007] Chen, D. J. , Osterrieder, N. , Metzger, S. M. , Buckles, E. , Doody, A. M. , Delisa, M. P. , & Putnam, D. (2010). Delivery of foreign antigens by engineered outer membrane vesicle vaccines. Proceedings of the National Academy of Sciences of the United States of America 107, 3099–3104 20133740 10.1073/pnas.0805532107 PMC2840271

[jev212066-bib-0008] Ellis, T. N. , & Kuehn, M. J. (2010). Virulence and immunomodulatory roles of bacterial outer membrane vesicles. Microbiol. Molecular Biology Reviews 74, 81–94 10.1128/MMBR.00031-09 PMC2832350 20197500

[jev212066-bib-0009] Fantappiè, L. , Irene, C. , De Santis, M. , Armini, A. , Gagliardi, A. , Tomasi, M. , Parri, M. , Cafardi, V. , Bonomi, S. , Ganfini, L. , Zerbini, F. , Zanella, I. , Carnemolla, C. , Bini, L. , Grandi, A. , & Grandi, G. (2017). Some Gram‐negative lipoproteins keep their surface topology when transplanted from one species to another and deliver foreign polypeptides to the bacterial surface. Molecular & Cellular Proteomics : MCP 16, 1348–1364 28483926 10.1074/mcp.M116.065094 PMC5500766

[jev212066-bib-0010] Gagliardi, A. , Lamboglia, E. , Bianchi, L. , Landi, C. , Armini, A. , Ciolfi, S. , Bini, L. , & Marri, L. (2016). Proteomics analysis of a long‐term survival strain of Escherichia coli K‐12 exhibiting a growth advantage in stationary‐phase (GASP) phenotype. Proteomics 16, 963–972 26711811 10.1002/pmic.201500314

[jev212066-bib-0011] Gerke, C. , Colucci, A. M. , Giannelli, C. , Sanzone, S. , Vitali, C. G. , Sollai, L. , Rossi, O. , Martin, L. B. , Auerbach, J. , Di Cioccio, V. , & Saul, A. (2015). Production of a Shigella sonnei vaccine based on generalized modules for membrane antigens (GMMA), 1790GAHB. PLoS One 10, e0134478. 26248044 10.1371/journal.pone.0134478 PMC4527750

[jev212066-bib-0012] Gerritzen, M. J.H. , Martens, D. E. , Wijffels, R. H. , Van Der Pol, L. , & Stork, M. (2017). Bioengineering bacterial outer membrane vesicles as vaccine platform. Biotechnology Advances 35, 565–574 28522212 10.1016/j.biotechadv.2017.05.003

[jev212066-bib-0013] Grandi, A. , Fantappiè, L. , Irene, C. , Valensin, S. , Tomasi, M. , Stupia, S. , Corbellari, R. , Caproni, E. , Zanella, I. , Isaac, S J. , Ganfini, L. , Frattini, L. , König, E. , Gagliardi, A. , Tavarini, S. , Sammicheli, C. , Parri, M. , & Grandi, G. (2018). Vaccination with a FAT1‐derived B cell epitope combined with tumor‐specific B and T cell epitopes elicits additive protection in cancer mouse models. Frontiers in Oncology 8, 481 30416985 10.3389/fonc.2018.00481 PMC6212586

[jev212066-bib-0014] Grandi, A. , Tomasi, M. , Zanella, I. , Ganfini, L. , Caproni, E. , Fantappiè, L. , Irene, C. , Frattini, L. , Isaac, S. J. , König, E. , Zerbini, F. , Tavarini, S. , Sammicheli, C. , Giusti, F. , Ferlenghi, I. , Parri, M. , & Grandi, G. (2017). Synergistic protective activity of tumor‐specific epitopes engineered in bacterial Outer Membrane Vesicles. Frontiers in Oncology 7, 1–12 29164053 10.3389/fonc.2017.00253 PMC5681935

[jev212066-bib-0015] Hisham, Y. , & Ashhab, Y. (2018). Identification of cross‐protective potential antigens against pathogenic Brucella spp. through combining pan‐genome analysis with reverse vaccinology. Journal of Immunology Research 2018, 1. 10.1155/2018/1474517 PMC6304850 30622973

[jev212066-bib-0016] Irene, C. , Fantappiè, L. , Caproni, E. , Zerbini, F. , Anesi, A. , Tomasi, M. , Zanella, I. , Stupia, S. , Prete, S. , Valensin, S. , König, E. , Frattini, L. , Gagliardi, A. , Isaac, S. J. , Grandi, A. , Guella, G. , & Grandi, G. (2019). Bacterial outer membrane vesicles engineered with lipidated antigens as a platform for Staphylococcus aureus vaccine. Proceedings of the National Academy of Sciences of the United States of America 116, 21780–21788 31591215 10.1073/pnas.1905112116 PMC6815149

[jev212066-bib-0017] Kaparakis‐Liaskos, M. , & Ferrero, R. L. (2015). Immune modulation by bacterial outer membrane vesicles. Nature Reviews. Immunology 15, 375–387 10.1038/nri3837 25976515

[jev212066-bib-0018] Keseler, I. M. , Mackie, A. , Santos‐Zavaleta, A. , Billington, R. , Bonavides‐Martínez, C. , Caspi, R. , Fulcher, C. , Gama‐Castro, S. , Kothari, A. , Krummenacker, M. , Latendresse, M. , Muñiz‐Rascado, L. , Ong, Q. , Paley, S. , Peralta‐Gil, M. , Subhraveti, P. , Velázquez‐Ramírez, D. A. , Weaver, D. , Collado‐Vides, J. , … Karp, P. D. (2017). The EcoCyc database: Reflecting new knowledge about Escherichia coli K‐12. Nucleic Acids Research. 45, D543–D550 27899573 10.1093/nar/gkw1003 PMC5210515

[jev212066-bib-0019] Kesty, N C. , & Kuehn, M J. (2004). Incorporation of heterologous outer membrane and periplasmic proteins into Escherichia coli Outer Membrane Vesicles. The Journal of Biological Chemistry 279, 2069–2076 14578354 10.1074/jbc.M307628200 PMC3525464

[jev212066-bib-0020] Klock, H. E. , & Lesley, S. A. (2009). The Polymerase Incomplete Primer Extension (PIPE) method applied to high‐throughput cloning and site‐directed mutagenesis. Methods in Molecular Biology (Clifton, N.J.) 498, 91–103 10.1007/978-1-59745-196-3_6 18988020

[jev212066-bib-0021] Kulp, A. J. , Sun, Bo , Ai, T. , Manning, A. J. , Orench‐Rivera, N. , Schmid, A. K. , & Kuehn, M. J. (2015). Genome‐wide assessment of outer membrane vesicle production in Escherichia coli. PLoS One 10, e0139200. 26406465 10.1371/journal.pone.0139200 PMC4583269

[jev212066-bib-0022] Kulp, A. , & Kuehn, M J. (2010). Biological functions and biogenesis of secreted bacterial outer membrane vesicles. Annual Review of Microbiology 64, 163–184 10.1146/annurev.micro.091208.073413 PMC3525469 20825345

[jev212066-bib-0023] Ladhani, S. N. , Ramsay, M. , Borrow, R. , Riordan, A. , Watson, J. M. , & Pollard, A. J. (2016). Enter B and W: Two new meningococcal vaccine programmes launched. Archives of Disease in Childhood 101, 91–95 26672098 10.1136/archdischild-2015-308928 PMC4717420

[jev212066-bib-0024] Malinverni, J. C. , & Silhavy, T. J. (2009). An ABC transport system that maintains lipid asymmetry in the Gram‐negative outer membrane. Proceedings of the National Academy of Sciences of the United States of America 106, 8009–8014 19383799 10.1073/pnas.0903229106 PMC2683108

[jev212066-bib-0025] Parikh, S. R. , Andrews, N. J. , Beebeejaun, K. , Campbell, H. , Ribeiro, S. , Ward, C. , White, J. M. , Borrow, R. , Ramsay, M. E. , & Ladhani, S. N. (2016). Effectiveness and impact of a reduced infant schedule of 4CMenB vaccine against group B meningococcal disease in England: A national observational cohort study. Lancet 388, 2775–2782 28100432 10.1016/S0140-6736(16)31921-3

[jev212066-bib-0026] Pileri, P. , Campagnoli, S. , Grandi, A. , Parri, M. , De Camilli, E. , Song, C. , Ganfini, L. , Lacombe, A. , Naldi, I. , Sarmientos, P. , Cinti, C. , Jin, B. , Grandi, G. , Viale, G. , Terracciano, L. , & Grifantini, R. (2016). FAT1: A potential target for monoclonal antibody therapy in colon cancer. British Journal of Cancer 115, 40–51 27328312 10.1038/bjc.2016.145 PMC4931367

[jev212066-bib-0027] Roier, S. , Zingl, F. G. , Cakar, F. , Durakovic, S. , Kohl, P. , Eichmann, T. O. , Klug, L. , Gadermaier, B. , Weinzerl, K. , Prassl, R. , Lass, A. , Daum, G. , Reidl, J. , Feldman, M. F. , & Schild, S. (2016). A novel mechanism for the biogenesis of outer membrane vesicles in Gram‐negative bacteria. Nature Communications 7, 10515. 10.1038/ncomms10515 PMC4737802 26806181

[jev212066-bib-0028] Rossi, O. , Caboni, M. , Negrea, A. , Necchi, F. , Alfini, R. , Micoli, F. , Saul, A. , Maclennan, C. A. , Rondini, S. , & Gerke, C. (2016). Toll‐Like receptor activation by generalized modules for membrane antigens from Lipid A mutants of Salmonella enterica Serovars Typhimurium and Enteritidis. Clinical and Vaccine Immunology : CVI 23, 304–314 26865597 10.1128/CVI.00023-16 PMC4820502

[jev212066-bib-0029] Serruto, D. , Bottomley, M. J. , Ram, S. , Giuliani, M. M. , & Rappuoli, R. (2012). The new multicomponent vaccine against meningococcal serogroup B, 4CMenB: Immunological, functional and structural characterization of the antigens. Vaccine 30, B87–B97. 22607904 10.1016/j.vaccine.2012.01.033 PMC3360877

[jev212066-bib-0030] Somerville, J. E. , Cassiano, L. , Bainbridge, B. , Cunningham, M. D. , & Darveau, R. P. (1996). A novel Escherichia coli lipid A mutant that produces an antiinflammatory lipopolysaccharide. The Journal of Clinical Investigation 97, 359–365 8567955 10.1172/JCI118423 PMC507025

[jev212066-bib-0031] Szklarczyk, D. , Gable, A. L. , Lyon, D. , Junge, A. , Wyder, S. , Huerta‐Cepas, J. , Simonovic, M. , Doncheva, N. T. , Morris, J. H. , Bork, P. , Jensen, L. J. , & Mering, C. V. (2019). STRING v11: Protein‐protein association networks with increased coverage, supporting functional discovery in genome‐wide experimental datasets. Nucleic Acids Research. 47, D607–D613 30476243 10.1093/nar/gky1131 PMC6323986

[jev212066-bib-0032] Van Der Pol, L. , Stork, M. , & Van Der Ley, P. (2015). Outer membrane vesicles as platform vaccine technology. Biotechnology Journal 10, 1689–1706 26912077 10.1002/biot.201400395 PMC4768646

[jev212066-bib-0033] Vorachek‐Warren, M. K. , Ramirez, S. , Cotter, R. J. , & Raetz, C. R.H. (2002). A triple mutant of Escherichia coli lacking secondary acyl chains on lipid A. The Journal of Biological Chemistry 277, 14194–14205 11830595 10.1074/jbc.M200409200

[jev212066-bib-0034] Zerbini, F. , Zanella, I. , Fraccascia, D. , König, E. , Irene, C. , Frattini, L F. , Tomasi, M. , Fantappiè, L. , Ganfini, L. , Caproni, E. , Parri, M. , Grandi, A. , & Grandi, G. (2017). Large scale validation of an efficient CRISPR/Cas‐based multi gene editing protocol in Escherichia coli. Microbial Cell Factories 16, 1–18 28438207 10.1186/s12934-017-0681-1 PMC5404680

