# Supplementary material for: Proteome‐minimized outer membrane vesicles from Escherichia coli as a generalized vaccine platform
Source: J Extracell Vesicles. 2021 Feb 16;10(4):e12066. doi: 10.1002/jev2.12066 (PMC7886703; doi:10.1002/jev2.12066)
Supplement: Supplementary file 1 — Supplementary information [file JEV2-10-e12066-s001.docx]

**Supplementary Information****
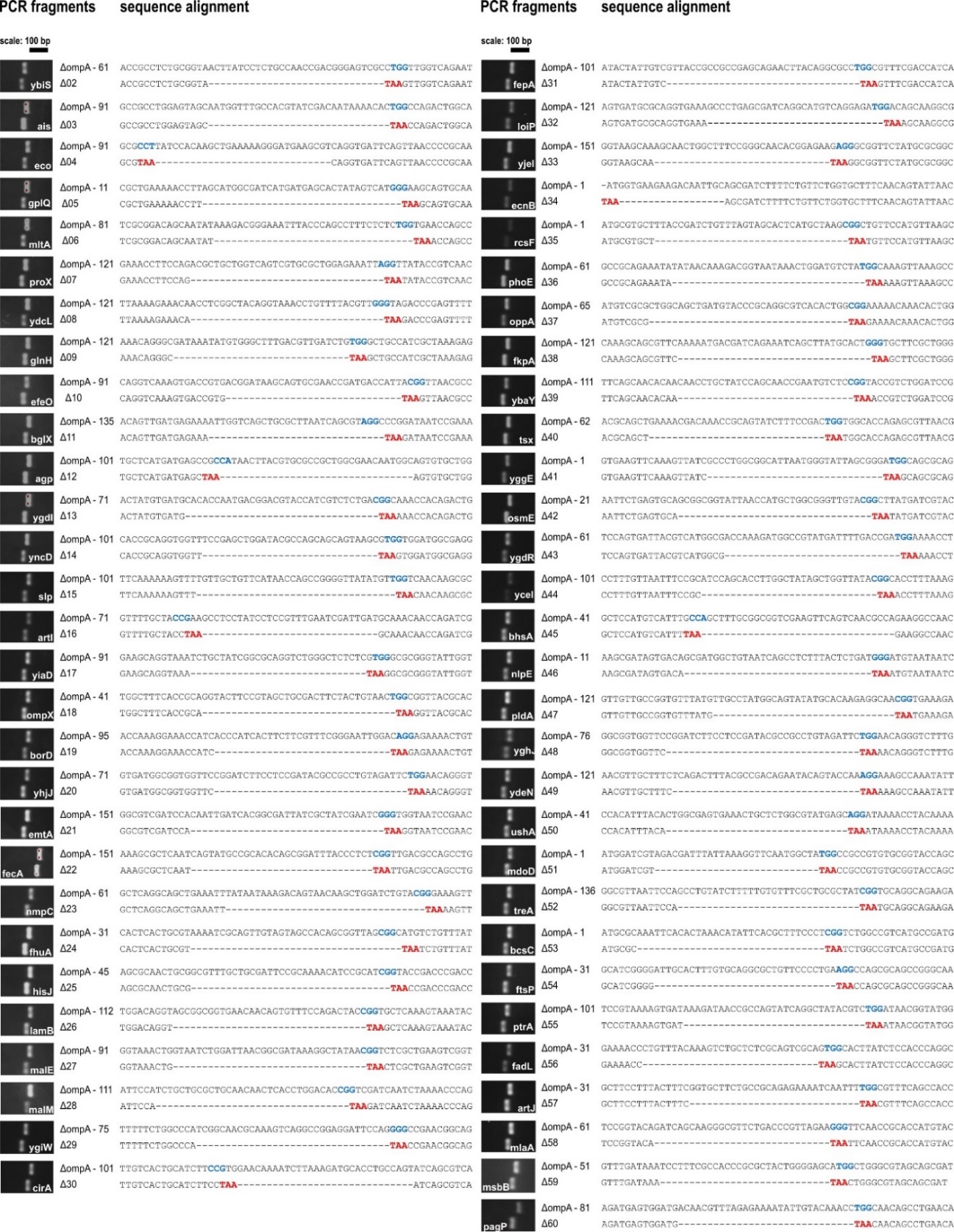
**

**Fig. S1. Sequence alignments of PCR amplified fragments spanning the targeted knockout region.** The PAM region identified from the progenitor *E. coli* BL21(DE3)*ΔompA* (*ΔompA*) is highlighted in blue. The alignment of the mutated region contains the deletion (-) and a stop codon (highlighted in red). Numbers indicated the position within the open reading frame of each gene.

**
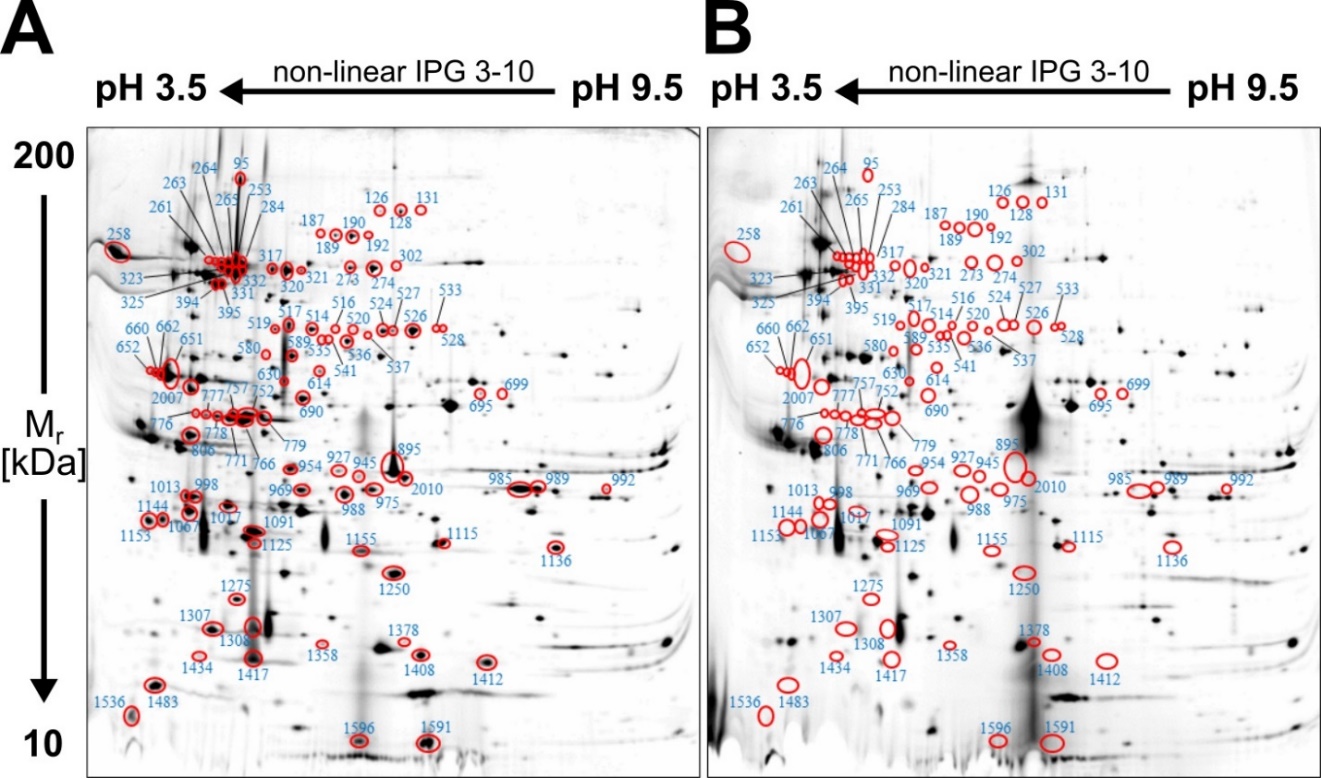
Fig. S2. Comparison of two-dimensional electrophoresis of OMVs*_ΔompA_* and OMVs*_Δ60_*.** Silver stained 2-DE gels of OMVs from *E. coli* BL21(DE3)*ΔompA* (**A**) and *E. coli* BL21(DE3)*Δ60* (**B**). The 2-DE maps are identical to the ones shown in Fig. 2, but all spots are additionally labelled with numbers to match with identified proteins listed in Table S2.


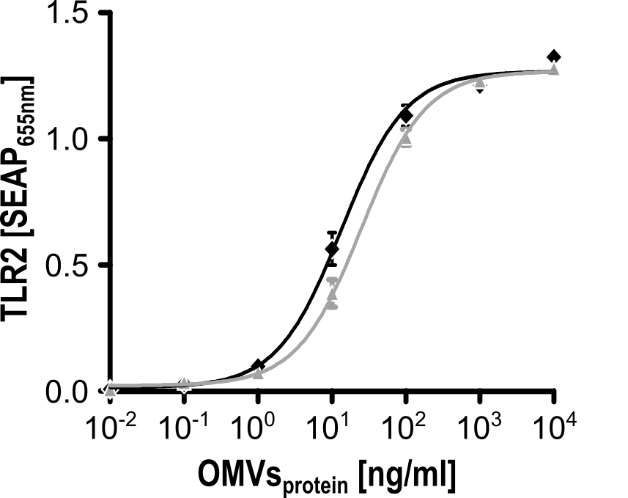


**Fig. S3.** *Analysis of TLR2 agonistic activity of OMVs -* HEK-Blue hTLR2 cells were incubated with different amounts of either OMVs*_ΔompA_* (♦) or OMVs*_Δ60_* (▲). After 17 hours the levels of secreted alkaline phosphatase (SEAP) upon hTLR2 stimulation by OMVs were determined by reading the absorbance of the culture supernatant at 655 nm.


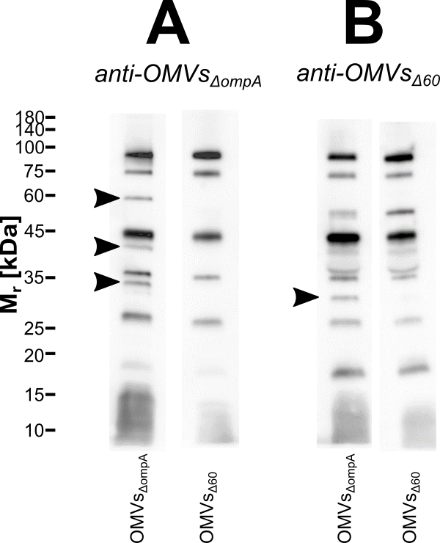


**Fig. S4. Immunogenicity of OMVs –** Ten μg of both OMVs*_Δ_*_ompA_ and OMVs*_Δ_*_60_ were analyzed by Western Blot using sera from mice immunized with either OMVs*_Δ_*_ompA_ (**A**) or OMVs*_Δ_*_60_ (**B**). Arrowheads indicate signals from proteins that are present in OMVs*_Δ_*_ompA_ but absent in OMVs*_Δ_*_60_ using the different sera.


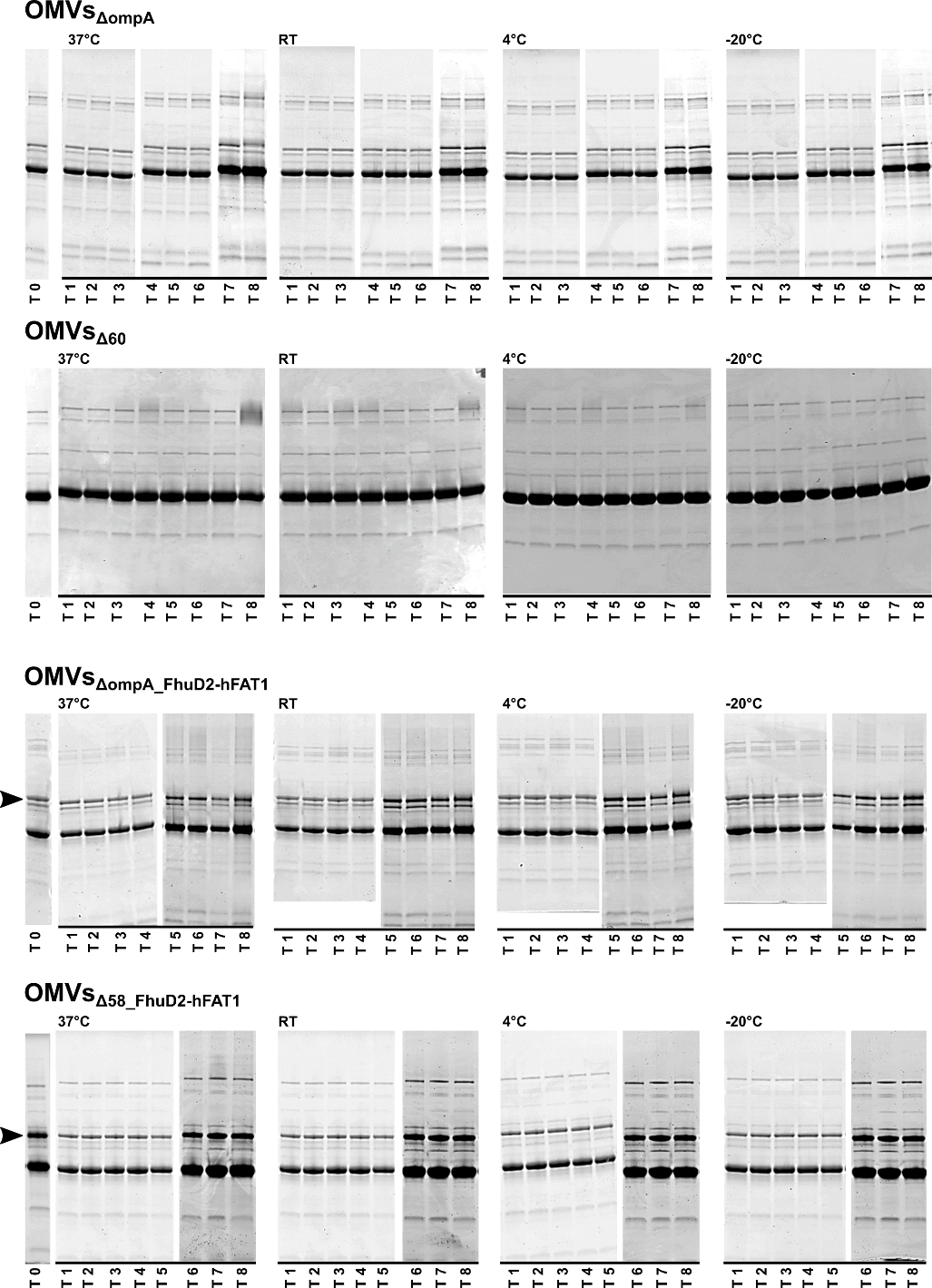


**Fig. S5. The stability of different OMV preparations at different temperatures.** The stability of OMVs*_ΔompA_*, OMVs*_Δ60_*, OMVs*_ΔompA_ FhuD2-hFAT1_* and OMVs*_Δ58_FhuD2-hFAT1_* was followed by SDS-PAGE over a period of three months. Aliquots were stored at 37°C, room temperature (RT), 4°C and -20°C and the amount corresponding to 10 μg total OMV proteins of the initial preparation were prepared in Laemmli buffer and boiled for subsequent analysis. The arrowhead indicates the heterologous antigen. T1: 1 day; T2: 3 days; T3: 7 days; T4: 14 days; T5: 21 days; T6: 4 weeks; T7: 2 months; T8: 3 months.

**
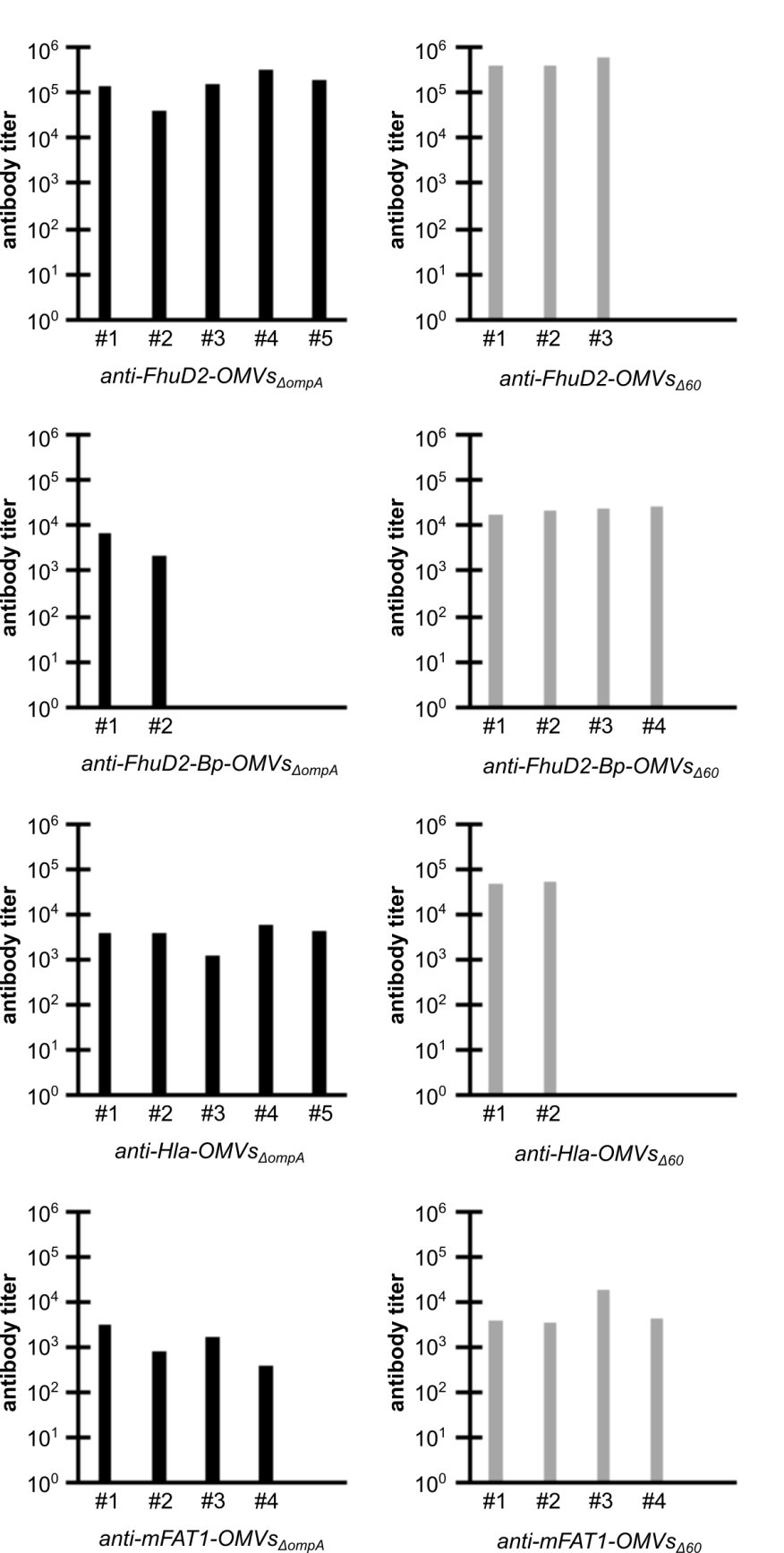
**

**Fig. S6. *IgG titers in single mice immunized with OMVs decorated with lipidated heterologous antigens (see also Fig. 5A)*.** Groups of 5 female BALB/c mice were immunized i.p. 3 times at two-week intervals with 2 μg of engineered OMVs*_Δ_*_ompA_ (black bars) and OMVs*_Δ_*_60_ (grey bars) in Alum. Sera were collected 7 days after the third immunization and IgG titers were measured by ELISA, coating the plates (200 ng/well) with either the corresponding purified recombinant antigen (FhuD2 and Hla_H35L_) or the corresponding synthetic peptide (Bp and mFAT1). ELISA titers was measured using available single mouse sera indicated on the x-axis (#1 - #5).
